# Current advance in bioconversion of methanol to chemicals

**DOI:** 10.1186/s13068-018-1265-y

**Published:** 2018-09-24

**Authors:** Wenming Zhang, Meng Song, Qiao Yang, Zhongxue Dai, Shangjie Zhang, Fengxue Xin, Weiliang Dong, Jiangfeng Ma, Min Jiang

**Affiliations:** 1 State Key Laboratory of Materials-Oriented Chemical Engineering, College of Biotechnology and Pharmaceutical Engineering, Nanjing Tech University, No. 30 Puzhu Road, Pukou District Nanjing, Nanjing, 211816 People’s Republic of China; 2 Jiangsu National Synergetic Innovation Center for Advanced Materials (SICAM), Nanjing Tech University, Nanjing, 211800 People’s Republic of China

**Keywords:** Methanol, Methylotrophs, Industrial biotechnology, Bio-based chemicals

## Abstract

Methanol has become an attractive substrate for biotechnological applications due to its abundance and low-price. Chemicals production from methanol could alleviate the environmental concerns, costs, and foreign dependency associated with the use of petroleum feedstock. Recently, a growing fraction of research has focused on metabolites production using methanol as sole carbon and energy source or as co-substrate with carbohydrates by native or synthetic methylotrophs. In this review, we summarized the recent significant progress in native and synthetic methylotrophs and their application for methanol bioconversion into various products. Moreover, strategies for improvement of methanol metabolism and new perspectives on the generation of desired products from methanol were also discussed, which will benefit for the development of a methanol-based economy.

## Background

Biotechnological production of fuels and chemicals has been considered as an alternative to petroleum-derived products [1]. In general, the cost of raw material accounts for most of total cost in biotechnological processes [2]. In white biotechnology, many low-cost raw materials, such as bagasse, sugar cane molasses and corn starch hydrolysates are widely used; however, the usage of carbohydrates derived from crops will competes with human demanding [3]. At present, lignocellulose has been proposed as a promising raw material for biosynthesis of fuels and chemicals, while several problems still exist, such as inefficient biocatalysts and high processing costs [3]. Thus, the adoption of low-price carbon substrates is still needed in the commercialization of industrial biotechnology [4].

Methanol, a non-food feedstock, has been considered as a next-generation carbon source due to its abundance and low-price [5]. Compared to other low-cost plant-derived feedstocks like molasses, methanol is purer and can be completely consumed in microbial metabolism. Degree of reduction per carbon of methanol is 6 while that of glucose is 4. Thus, more electrons are available, which can enhance the yield of biofuels and chemicals [6]. Furthermore, the use of methanol reduces the risk of contaminations, and residual methanol can easily be removed by elevated temperature [7]. Importantly, methanol price continues to slide due to the maturity of the methanol production process [8]. Therefore, biosynthesis of chemicals from methanol has attracted much interest, representing a promising process [9].

**Fig. 1:**
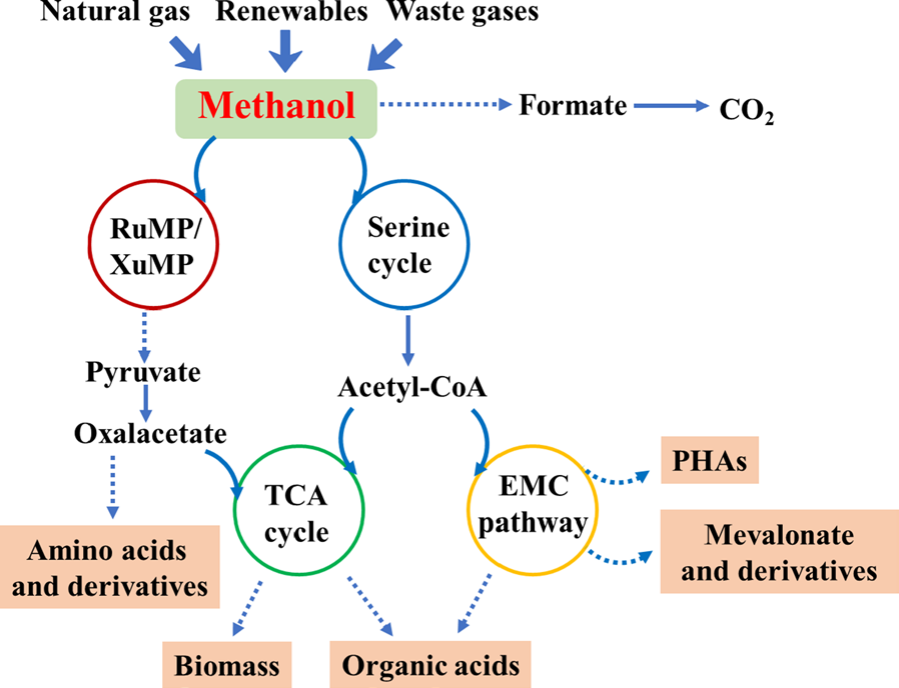
Schematics of various of chemicals production from methanol. Methanol obtained from different resources can be oxidized to CO_2_ by dissimilation or enter the carbon metabolic pathways and converted into chemicals via certain assimilation pathways. Solid arrows represent one step reactions, while dotted arrows show multi-step reactions

Methylotrophs, which can utilize reduced C1 compounds as sole carbon source for growth, have been used for the production of various valuable chemicals (Fig. 1). In nature, methylotrophs can be divided into two groups, methylotrophic bacteria and yeasts. To overcome inefficient genetic-transfer systems and low yields of desirable metabolites, synthetic methylotrophy has become an increasing attention. Within this review, we summarized the current significant progress using native and synthetic methylotrophy and their use for methanol bioconversion into various products (Table 1). In addition, some strategies for improvement of methanol metabolism and production of more value-added products were discussed.

**Table 1:** Various of chemicals production using native or synthetic methylotrophs

Substance class	Product	Concentration	Yield	Organism	Reference
PHAs	PHB	40–52.9 g/L	0.09–0.12 g/g	*M. extorquens* ATCC 55366	[16]
		9.5 g/L	0.16 g/g	*M. extorquens* DSMZ 1340	[18]
		136 g/L	0.18 g/g	*Pseudomonas* sp. K	[90]
	PHB (8000 –10,000 kDa)	7.04 g/L	0.17 g/g	*M. halotolerans C2*	[17]
	PHB-*co*-HV	~ 1 g/L	0.17 g/g	*Methylobacterium* sp. strain GW2	[21]
	PHB-*co*-HV	2.34 g/L	–	*Methylocystis* sp.* WRRC1*	[91]
Amino acids	l-Glutamate	38.8 g/L	~ 0.13 g/g	*M. glycogenes*	[25]
		55 g/L	0.36 g/g	*Bacillus methanolicus* MGA3	[27, 28]
	l-Threonine	11 g/L	–	*M. glycogenes* AL119	[25]
	l-Lysine	8 g/L	–	Introduction of the *dapA* gene encoding dihydrodipicolinate synthase into *M. glycogenes AL119*	[26]
		35 g/L	–	*B. methanolicus* MGA3 mutant 13A52-8A66	[27]
		11.3 g/L	–	Expressed l-lysine/l-arginine exporter (LysE) in *M. methylotrophus*	[30]
	l-Serine	54.5 g/L	8.3% from methanol, 39.3% from glycine	*M. extorquens*	[31]
		65 mg/mL	0.62 g/g	*Methylobacterium* sp. MN43	[32]
Organic acids	Pyruvate	0.26 g/L	0.25 g/g	The methanol metabolic pathways originating from *P. pastoris* were constructed into the chromosome of *S. cerevisiae*	[2]
	Mesaconic	70 mg/L	0.0175 g/g	Expressed thioesterase *yciA* in *M. extorquens*	[44]
	Methylsuccinic acid	60 mg/L	0.015 g/g	Expressed thioesterase *yciA* in *M. extorquens*	[44]
Fine chemicals	Mevalonate	2.67 g/L	0.085 g/g	Biosensor-assisted transcriptional regulator engineering used for *M. extorquens*	[54]
	α-Humulene	1.65 g/L	0.031 g/g	Expressed α-humulene synthase and farnesyl pyrophosphate synthase in *M. extorquens*	[56]
	Naringenin	3.5 mg/L	4.7% ^13^C-labeling in naringenin	The pathways for methanol assimilation and naringenin synthesis were introduced into *E. coli*	[60]
	Monacolin J	593.9 mg/L	0.35 mg/g	The biosynthetic pathways for monacolin J and lovastatin were assembled into *P. pastoris*	[65]
	Lovastatin	250.8 mg/L	0.15 mg/g	The biosynthetic pathways for monacolin J and lovastatin were assembled into *P. pastoris*	[65]
Other chemicals	Cadaverine	11.3 g/L	–	Expressed lysine decarboxylase in *B. methanolicus*	[67]
		1.5 g/L	–	The pathways for methanol assimilation and lysine decarboxylase were introduced into *C. glutamicum*	[64]
	γ-Aminobutyric acid	9 g/L	–	Expressed glutamate decarboxylase in *B. methanolicus* MGA3	[77]
Proteins	Green fluorescent protein (GFP)	4 g/L	0.3 g/g	Expressed green fluorescent protein in *M. extorquens*	[82]
	Enterocin P	155 ng/mL	–	Expressed the Enterocin P structural gene in *M. extorquens*	[84]
	Cry1Aa	1.26 mg/L	9 mg/g DCW, 4.5% of total protein	Engineered *M. extorquens*	[85]

### Polyhydroxyalkanoates

Microbial synthesis of polyhydroxyalkanoates (PHAs) as a potential sustainable replacement for non-degradable petroleum-based plastics has attracted wide attention due to their biocompatibility and biodegradability [10, 11]. PHAs are naturally produced by bacteria and archaea from various carbon sources, such as saccharides, *n*-alkanes, *n*-alcohols and gases [12]. Based on the advantages of methanol as substrate, the application of methylotrophs is regarded as a promising way for biosynthesis of PHAs from methanol [13]. Recently, many efforts have been made to enhance the PHAs content and control the PHA structures to form homopolymers, random copolymers and block copolymers for diversity of material properties [14]. *Methylobacterium extorquens* was able to use methanol as sole carbon and energy source for the production of poly-3-hydroxybutyrate (PHB). *M. extorquens* AM1 has been studied for PHB production more than 30 years, and produced the PHB titer to 149 g/L [15] *M. extorquens* ATCC 55366 accumulated PHB up to 46%, while PHB yield was only 0.09 g/g methanol. Moreover, higher molecular weight PHB (900–1800 kDa) will be produced under methanol-limiting conditions (< 0.01 g/L) [16]. Media components optimization was an effective strategy for cell growth and PHB production. When 0.12 g/L of NH_4_^+^ was added, *Methyloligella halotolerans* C2 could produce 7.04 g/L of 8000–10,000 kDa PHB [17]. Under a deficiency of nitrogen and magnesium condition, 62.3% of DCW and 9.5 g/L of PHB with yield of 0.16 g/g were generated by *M. extorquens* DSMZ 1340 [18].

Due to the limited rigidity and brittleness of the PHB structure, further studies were focused on functionalized PHAs containing C–C double bonds produced when unsaturated carboxylic acids were used as an auxiliary carbon source [19, 20]. The co-polyester poly-3-hydroxybutyrate-poly-3-hydroxyvalerate (PHB/HV) was accumulated in *Methylobacterium* sp. strain GW2, using valeric acid as co-substrates. Moreover, an average molecular mass of biopolymers was 229–233 kDa for PHB and 362–411 kDa for PHB/HV [21]. *M. extorquens* AM1 was able to synthesize P(3HB-*co*-3HV) copolymers from methanol as a sole carbon source under cobalt-limited conditions. After genetic engineered, a recombinant strain could produce a PHA terpolymer composed of 3HB, 3HV, and a C6-monomer, (*R*)-3-hydroxyhexanoate, indicating it was possible to produce practical PHA copolymers by methylotrophic bacteria using methanol as a feedstock [13]. Due to the cheaper cost of methanol compared to pure sugar substrates, the use of methylotrophs for the production of PHA could reduce PHA cost. However, the content of PHA from methanol (46% cell dry mass) is lower than that from sugars (50–88% cell dry mass) [19]. Moreover, methanol concentration was no more than 100 mM during fermentation, and the tolerance of strain to methanol should be improved for the industrial application. Moreover, further study is needed to improve the content and yield of PHA before industrialized production.

### Amino acids

Amino acids are widely used as food and feed additives, flavor enhancers, pharmaceuticals and polymer materials [22, 23]. At present, microbial fermentation is still the primary way for large scaling amino acid production, in which the main producer is *Corynebacterium glutamicum* [24]. To reduce production cost, many cheap substrates, such as molasses and starch were developed for the production of amino acids. In parallel, some microorganisms utilizing more inexpensive and non-food substrates were explored to reduce production cost [23].

Many native and engineered methylotrophs were used to overproduce different amino acids including glutamate, lysine, serine, and threonine from methanol [25–32]. For example, *Methylobacillus glycogenes* RV3 can produce 38.8 g/L of l-glutamate. After chemical mutagenized with *N*-methyl-*N*′-nitro-*N*-nitrosoguanidine (NTG), a mutant AL119 can secret 11 g/L of l-threonine, and mutant DHL 122 can produce 3.1 g/L of l-lysine and 5.6 g/L of l-threonine [25]. When overexpressed a dihydrodipicolinate synthase from *M. glycogenes*, 37 g/L of glutamate and 8 g/L of lysine were accumulated in *M. glycogenes* DHL122 at 37 °C [26]. The methylotrophic bacterium *Bacillus methanolicus* is a promising candidate for amino acid production from methanol. Because genes involved in methanol metabolism are plasmid-encoded, which can be upregulated in cells utilizing methanol, *B. methanolicus* MGA3 has high methanol consumption rate. It can produce 59 g/L of l-glutamate and 0.4 g/L of l-lysine at 50 °C in fed-batch cultivation, using methanol as carbon source [27, 28]. Due to the lack of efficient genetic tools, random chemical mutagenesis was the main method for strains engineering [24, 29]. For example, 11 g/L of l-lysine and 69 g/L of l-glutamate were accumulated in an *S*-(2-aminoethyl)-cysteine-resistant mutant M168-20 [28]. A homoserine dehydrogenase mutant, 13A52-8A66 was found to secret 35 g/L of l-lysine and 28 g/L of l-glutamate [27].

An excretion transport system is crucial to amino acids production. When the l-lysine/l-arginine exporter (LysE) from *C. glutamicum* was expressed in *Methylophilus methylotrophus*, the production of l-lysine was improved to 11.3 g/L, suggesting that amino acid exportation systems would facilitate amino acid production [30]. Due to the serine cycle, *M. extorquens* can produce serine from methanol and glycine. In resting cells of *M. extorquens*, 54.5 g/L of l-serine was achieved under appropriate conditions. The yield can be up to 8.3% from methanol and 39.3% from glycine, respectively [31]. Similarly, 65 mg/mL of l-serine were obtained from 104 mg/mL of methanol and 50 mg/mL of glycine in resting cells of *Methylobacterium* sp. MN43 when l-serine degradation was blocked [32].

Substrate consumption rate and product yield are important indicators for microbial production processes. It was reported that cell growth rate and methanol consumption rate can up to 0.4/h and 7 g/(L h) in *B. methanolicus* MGA3, which is close to cell growth rate (0.53/h) and glucose consumption rate [6.6 g/(L h)] in *C. glutamicum* [7, 33]. Moreover, the theory yield of l-lysine from methanol (0.71 g/g) by *B. methanolicus* is the same as that from glucose (0.78 g/g) by *C. glutamicum* [23]. Importantly, *B. methanolicus* are able to grow at temperatures from 35 to 60 °C with a growth optimum around 50–55 °C [34]. Based on the above advantages, biosynthesis of amino acids from methanol by *B. methanolicus* represents a promising and sustainable method.

### Organic acids

Organic acids, especially for low molecular weight carboxylic acids, have been widely applied in food, pharmaceutical, cosmetic, detergent, polymer, and textile industry [35]. In view of the crisis of fossil fuels and concerns of environmental pollution, more attention has been given to the production of organic acids via fermentation using renewable feedstocks. In the last decade, several organic acids were produced via biorefinery using various types of microorganisms, such as succinic acid production by *E. coli* [36, 37] or *Yarrowia lipolytica* [38, 39], malic acid production by *Aspergillus oryzae* [40, 41], citric acid production by *Aspergillus niger* [42] and itaconic acid production by *Aspergillus terreus* [43]. To realize industrial production and make the bioprocess more economical, adoption of inexpensive feedstock for organic acids production offers a promising alternative. Based on advantages of abundant sources, low-cost, highly reduced and non-food substance, methanol has gained more attractive attention for biotechnological processes.

**Fig. 2:**
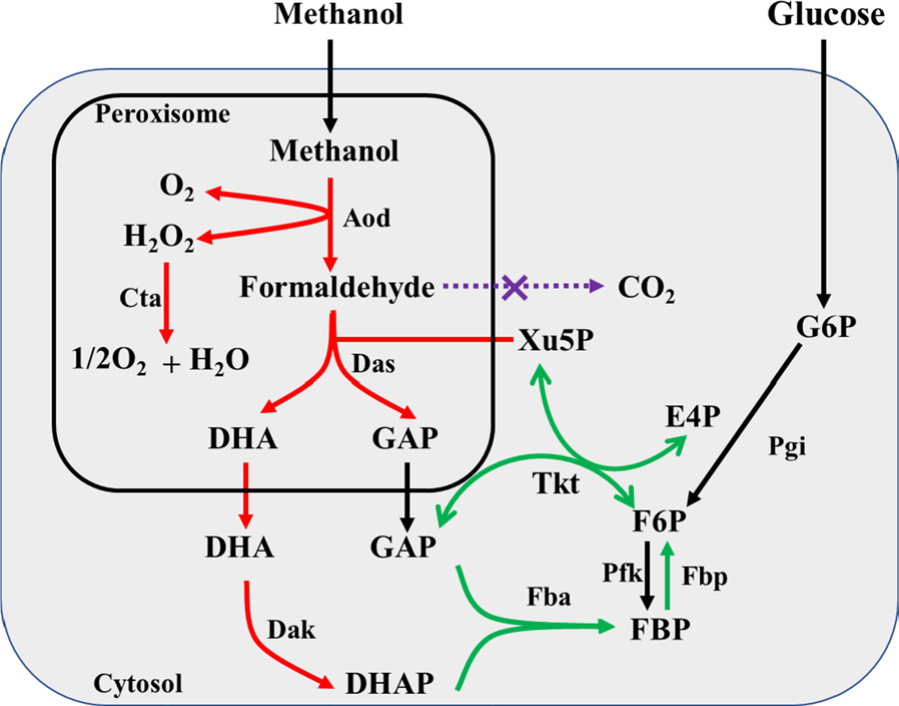
Strategies to enhance the metabolic flux from methanol to metabolites. Methanol is first oxidized to formaldehyde by peroxisomal enzyme alcohol oxidase, which is assimilated via the xylulose 5-phosphate pathway (XuMP) (red lines). Formaldehyde dissimilation pathway is blocked (purple line). The regeneration of xylulose 5-phosphate is improved by overexpressed genes of pentose phosphate pathway (green lines). Enzymes: Aod, alcohol oxidase; Cta, catalase; Das, dihydroxyacetone synthase; Dak, dihydroxyacetone kinase; Tkt, transketolase; Fba, fructose-bisphosphate aldolase; Pfk, phosphofructokinase; Fbp, fructose bisphosphatase; Pgi, phosphoglucose isomerase. Metabolites, DHA, dihydroxyacetone; DHAP, dihydroxyacetone phosphate; GAP, glyceraldehyde 3-phosphate; F6P, fructose 6-phosphate; FBP, fructose 1,6-bisphosphate; Xu5P, xylulose 5-phosphate; E4P, erythrose 4-phosphate; G6P, glucose 6-phosphate

*Saccharomyces cerevisiae* was metabolic engineered to achieve pyruvate production from methanol by expressing the methanol metabolic module originated from *Pichia pastoris* [2]. The results showed that 0.26 g/L of pyruvate with a yield of 0.25 g/g was achieved from methanol. The biomass was increased 3.13% compared to the wild-type strain. Moreover, the consumption of methanol was improved when 1 g/L of yeast extract was supplemented. Given that, some dicarboxylic acids, such as malic acid and succinic acid, could be also produced from methanol if the enzymes of reductive tricarboxylic acid (rTCA) pathway were introduced into this strain. However, methanol utilization rate and metabolites production should be improved by the application of available genetic tools, such as harboring inducible promoters and balancing the cooperation of enzymes. It’s worth noting that methanol is first oxidized to formaldehyde by methanol dehydrogenase, while formaldehyde dissimilation into CO_2_ is ubiquitous in organisms. Therefore, to enhance the metabolic flux from methanol to metabolites, formaldehyde dissimilation should be blocked. Furthermore, the regeneration of xylulose 5-phosphate is crucial to sustain the pathway running (Fig. 2).

Some uncommon dicarboxylic acids, such as ethylmalonic acid, methylsuccinic acid, mesaconic acid and methylmalic acid, can also be produced from methanol [44]. The ethylmalonyl-CoA pathway (EMCP), harboring several saturated or unsaturated carboxylated C4- and C5-acyl-CoA esters, was discovered in the methylotroph *M. extorquens* AM1 [45, 46]. YciA, an acyl-CoA thioester hydrolase, was heterologously expressed in *M. extorquens* AM1, leading to production of 70 mg/L mesaconic and 60 mg/L methylsuccinic acid from methanol [44]. This work provides the possibility of EMCP-derived dicarboxylic acids production by other thioesterases.

### Fine chemicals

Terpenoids and sesquiterpenoids are widely used for drugs, flavors and fragrances, representing a large class of natural products, such as sterols and carotenoids [47–49]. In nature, they are produced by the mevalonate (MVA) pathway or methylerythritol 4-phosphate (MEP) pathway [50]. In the MVA pathway, two molecules of acetyl-CoA were converted into acetoacetyl-CoA by acetoacetyl-CoA synthase, which is naturally present in *M. extorquens*. This feature suggests that *M. extorquens* is a promising candidate for terpenoid and derivative biosynthesis, because it is difficult to heterologous express an active acetoacetyl-CoA synthase in chassis organism [51]. In addition, since no native MVA pathway exists, a heterologous MVA pathway will not be interfered by endogenous regulation [52].

To achieve mevalonate production, a natural operon and an artificial operon employing key enzymes of the MVA pathway were introduced into *M. extorquens* AM1, resulting in 56 and 66 mg/L of mevalonate obtained in shake flask fermentations, respectively. Increasing flux of the precursor acetyl-CoA towards acetoacetyl-CoA by expressing *phaA* gene (encoding acetoacetyl-CoA thiolase) from *Ralstonia eutropha* achieved 80 mg/L of mevalonate. Further regulation of *phaA* expression using different strengths RBSs (ribosomal binding sites) produced 215 mg/L of mevalonate. Finally, 2.22 g/L of mevalonate with a yield and productivity of 28.4 mg/g and 7.16 mg/(L h) was obtained in fed-batch fermentation, representing the first reported de novo synthesized mevalonate from methanol [49]. To further decrease the cost and improve the titer, the medium was optimized by changing the concentration of phosphate and trace elements. The mevalonate production reached 340 mg/L using the optimized medium. In 5 L fed-batch fermentation, mevalonate concentration, yield and productivity reached 2.59 g/L, 48.90 mg/g and 12.33 mg/(L h), respectively [53]. It is known that acetyl-CoA is a key precursor for the synthesis of mevalonate. To increase carbon metabolic flux into acetyl-CoA for mevalonate synthesis, a biosensor-assisted transcriptional regulator QscR was used to control metabolic flux re-distribution. The results of ^13^C-labeling suggested that acetyl-CoA flux was improved by 7%. Further transcriptional analysis revealed that NADPH generation and *fumC* (encoding fumarase C) overexpression were two main factors for the metabolic flux re-distribution. Finally, 2.67 g/L of mevalonate with yield of 0.085 g/g was obtained by fed-batch fermentation, representing the highest yield in engineered *M. extorquens* AM1 [54]. This strategy provides not only a method for the effective synthesis of mevalonate, terpenoids and other derivatives, but also a new way for the refinery and improvement of the production of other products based on acetyl-CoA, such as fatty acids and derivatives.

α-Humulene, a monocyclic sesquiterpenoid, has raised wide attention due to its anti-inflammatory and potential anti-cancer properties [55]. For production of α-humulene, α-humulene synthase and farnesyl pyrophosphate (FPP) synthase were heterologously expressed in *M. extorquens* AM1 [56]. The engineered strain produced 18 mg/L of α-humulene, representing the first synthesis of α-humulene from methanol. When a prokaryotic MVA pathway from *Myxococcus xanthus* together with RBS optimized α-humulene synthase and FPP synthase were introduced into AM1, the engineered strain produced 58 mg/L of α-humulene. To further reduce the flux of by-product carotenoid from FPP, a diapolycopenedial formation deficient strain was used [57], resulting in 1.65 g/L of α-humulene with yield of 0.031 g/g produced in fed-batch fermentation, representing the highest concentration for de novo synthesis of α-humulene to date.

Besides native methylotrophs, the concept of synthetic methylotrophy has attracted much interest. Recently, efforts towards achieving methanol utilization were reported in *E. coli* [58–62] and *Corynebacterium glutamicum* [63, 64]. And more and more chemicals have been synthesized from methanol in these platform microorganisms [60–64]. The flavanone naringenin is the first example of methanol bioconversion in methylotrophic *E. coli* [60]. Whitaker et al. expressed methanol dehydrogenase (Mdh) from *B. stearothermophilus* in combination with ribulose monophosphate (RuMP) pathway enzymes from *B. methanolicus* for methanol utilization in *E. coli*. ^13^C-labeling showed that the intermediate in glycolytic, TCA cycle and some amino acids can be labeled; demonstrating methanol was assimilated by the recombinant *E. coli*. To demonstrate that methanol was able to be converted to chemicals, the naringenin biosynthesis pathway was incorporated into methylotrophic *E. coli* strain. The results revealed up to 4.7% ^13^C-labeling in naringenin and 18% of the total naringenin pool contained at least one carbon labeled. Importantly, they firstly found the methanol assimilation can be improved during growth with small amounts of yeast extract. To further elucidate the mechanism, ^13^C-tracers experiments were used to examine intracellular metabolites during methanol assimilation [61]. Twenty five potential co-substrates were evaluated, indicating threonine was the optimum co-substrate for methanol assimilation. Then the threonine degradation pathway was improved by deletion of the repressor Lrp, which led to the improvement of biomass [61].

Recently, it was reported that a methylotrophic yeast was used to produce pharmaceuticals from methanol. The biosynthesis module of monacolin J and lovastatin were assembled in *Pichia pastoris* using methanol as the sole carbon source, leading to 60.0 mg/L of monacolin J and 14.4 mg/L of lovastatin production in pH-controlled monoculture [65]. To relieve imbalance metabolism and metabolic stress in monoculture, the pathways were split and re-distributed in a *P. pastoris*–*P. pastoris* consortium. The results proved that production of monacolin J and lovastatin were improved by 55% and 71%. Finally, 593.9 mg/L of monacolin J and 250.8 mg/L of lovastatin were obtained in bioreactor fermentations. This work not only provides the feasibility of a methylotrophic yeast for production of pharmaceuticals, but also offers a new view to produce chemicals from methanol using co-culture system. Microbial co-cultures have the ability to reduce metabolic burden, enhance systematic robustness and improve target chemicals production [66]. For example, one strain used to convert methanol to intermediate metabolites, followed by its conversion to other products by the other strain. Importantly, the functions of microbial consortia should be optimized, including optimization of metabolite exchange and control of molecular signaling pathways, electron transfer, and cofactor.

### Other chemicals

Besides the aforementioned chemicals, some value-added chemicals can also obtain from methanol by native and synthetic methylotrophs, such as cadaverine and γ-aminobutyric acid. The diamine 1,5-diaminopentane (cadaverine), a monomeric polyamide building block, is an important platform chemical used for production of various polyamides and mainly manufactured by petroleum-based raw materials [67]. Facing the threat of oil depletion and environmental pollution, many efforts have been made for improving the production process of polyamines from renewable resources [68]. Cadaverine has been generated from various raw materials, such as starch or hemicellulose through biological fermentation [69, 70]. Recently, methanol-based cadaverine can also be produced by methylotrophic or non-methylotrophic organisms. Although cadaverine is a non-native product, it can be obtained from lysine decarboxylation by lysine decarboxylase. The facultative methylotrophic bacterium *B. methanolicus* is a great candidate for l-lysine overproduction, because the maximum theoretical yield can reach 0.82 g/g from methanol, which is comparable to the maximum yield from glucose in *C. glutamicum* [23, 71]. Two different lysine decarboxylase genes *cadA* and *ldcC* from *E. coli* were introduced into *B. methanolicus*. Results showed that strains expressing *cadA* produced 0.5 g/L of cadaverine in shake flask conditions. Finally, the production of cadaverine can reach up to 11.3 g/L from methanol in high density fermentation [67]. *C. glutamicum* can produce 120 g/L lysine in fed-batch fermentation [72], representing a promising candidate for cadaverine production. After heterologous expressed *cadA* encoding lysine decarboxylase from *E. coli*, the engineered *C. glutamicum* produced 2.3 g/L of cadaverine with yield of 0.17 g/g [73]. Synthetic methylotrophic *C. glutamicum* was also able to produce cadaverine using methanol as co-substrate [64]. Methanol dehydrogenase and RuMP pathway enzymes from *B. methanolicus* and lysine decarboxylase LdcC from *E. coli* were employed for methanol utilization and cadaverine synthesis in *C. glutamicum*, respectively. 1.5 g/L of cadaverine was obtained in shake flask fermentation when methanol was used as an auxiliary substrate [64]. ^13^C-labeling experiments revealed that labeling of cadaverine was detected; indicating ^13^C-methanol was indeed converted to cadaverine by methanol assimilation. However, compared with methylotrophic bacterium *B. methanolicus*, synthetic methylotrophic *C. glutamicum* cannot sustain growth on methanol alone in a defined medium. Moreover, only about 5% labeled carbon was observed when used minimal medium containing 50 mM glucose and 50 mM ^13^C-methanol [64]. Therefore, further studies are still needed to increase titer and yield by synthetic biology or process design.

γ-Aminobutyric acid (GABA), a non-protein amino acid, has been widely studied due to its numerous physiological and pharmacological functions. The biosynthesis of GABA is the decarboxylation of l-glutamate by glutamate decarboxylase (GAD), which has been found in bacteria, plants and insects [74]. In recent years, several GABA-producing strains were isolated or engineered for the efficient production of GABA [75, 76]. Methanol-based GABA production was also achieved by engineering methylotrophic *B. methanolicus* [77]. Two glutamate decarboxylase genes *gadSt* (from *Sulfobacillus thermosulfidooxidans*) and *gadB* (from *E. coli*) were heterologously expressed in *B. methanolicus*, respectively. From the shake flask experiments, the strain expressing *gadB* produced higher GABA (0.4 g/L) than that of the strain expressing *gadSt* (0.03 g/L). However, 29 g/L of l-glutamate and 0.1 g/L of GABA were accumulated in methanol fed-batch fermentation, indicating glutamate decarboxylase was inactive. After two-phase fermentation strategy and the pH shift were employed, the final production of GABA was increased to 9 g/L, which provides a new process of production of GABA and its derivates, such as biodegradable plastic polyamide 4.

### Proteins

After World War II, technological advances in biotechnology led to the idea of developing single-cell proteins (SCP) using microorganisms, such as algae, bacteria and yeasts [78]. SCP possesses a high nutritional value, in which amino acid composition was similar to that of fish meal. Thus, SCP was usually used as animal feed and supplemental feedstuff material [79]. In the early 1970s, SCP production from methanol was studied intensively [78, 80]. Based on these advantages, a series of large-scale SCP production from methanol was established using high-cell-density fermentation strategy. A pilot plant equipped with an airlift fermentor (20 m^3^) was constructed by Mitsubishi Gas Chemical Company (MGC) for SCP production using methylotrophic bacteria in 1974 [81]. Imperial Chemical Industries (ICI) also constructed a 1500 m^3^ airlift reactor in the 1970s and 1980s [70]. When SCP is used as the source of animal feed protein, two factors are crucial: safety and nutritional value. Compared to methylotrophic bacteria, methylotrophic yeasts are safer without toxin formation. However, the protein content is lower than in bacteria. Moreover, the content of some amino acids is limited, such as methionine. Therefore, genetic and metabolic engineering should be adopted to obtain higher protein or amino acid contents in methanol-utilizing yeast.

Methylotrophs have also been used for production of various proteins, such as enzymes, antibodies and hormones. Green fluorescent protein (GFP) was usually used as a model protein to study the efficiency of vectors and promoters. Based on GFP production, the ability of two different expression vectors (pRK310 and pCM110) and promoters (Plac and PmxaF) for heterologous protein expression by *M. extorquens* was determined [82]. Results suggested that clones harboring PmxaF-GFP-pCM110 produced approximately 100-fold more GFP than those harboring Plac-GFP-pRK310 in shake flask experiments. The maximum growth rate and the biomass yield were 0.18 h^−1^ and 0.3 g/g in fed-batch fermentation. Finally, 4 g/L of GFP with yield of 80 mg/g was obtained using the recombinant harboring PmxaF-GFP-pCM110 [82], indicating *M. extorquens* might be a promising candidate for overexpression of recombinant proteins.

Enterocin P (EntP), a new pediocin-like bacteriocin, is produced by *Enterococcus faecium* strains. Based on a broad-spectrum antimicrobial property, EntP has a potential application as an effective food antimicrobial agent [83]. However, many enterococci contain potential virulence factors; thus, heterologous production of bacteriocins in safer hosts should be developed. Recently, *M. extorquens* was engineered for the production of EntP using methanol as the sole carbon and energy source [84]. 155 ng/mL of EntP was obtained in the recombinant, indicating an improvement of 25-fold increase than *E. coli* on EntP production. Importantly, the purification of EntP was biologically active [84]. This study provides a new method for production of bacteriocins and proteins of interest from methanol by methylotrophs.

Cry1Aa is an insecticidal protein from *Bacillus thuringiensis*, which cannot colonize foliage and propagate vegetative. Thus, *cry1Aa* gene from *B. thuringiensis* was heterologously expressed in a plant-colonizing methylotroph, *M. extorquens* [85]. Bipyramidal intracellular crystal-shaped inclusions were the same as the crystalline inclusions produced by *B. thuringiensis*. Comparisons of biomass and growth rate of wild-type and recombinant strains indicated that the production of recombinant protein does not influence the growth performance of the *M. extorquens* strain. However, just 4.5% of total protein was obtained, which is lower than that of other microorganisms (10–20%), indicating more efforts are needed to increase the production of Cry1Aa in *M. extorquens*.

### Future perspectives

**Fig. 3:**
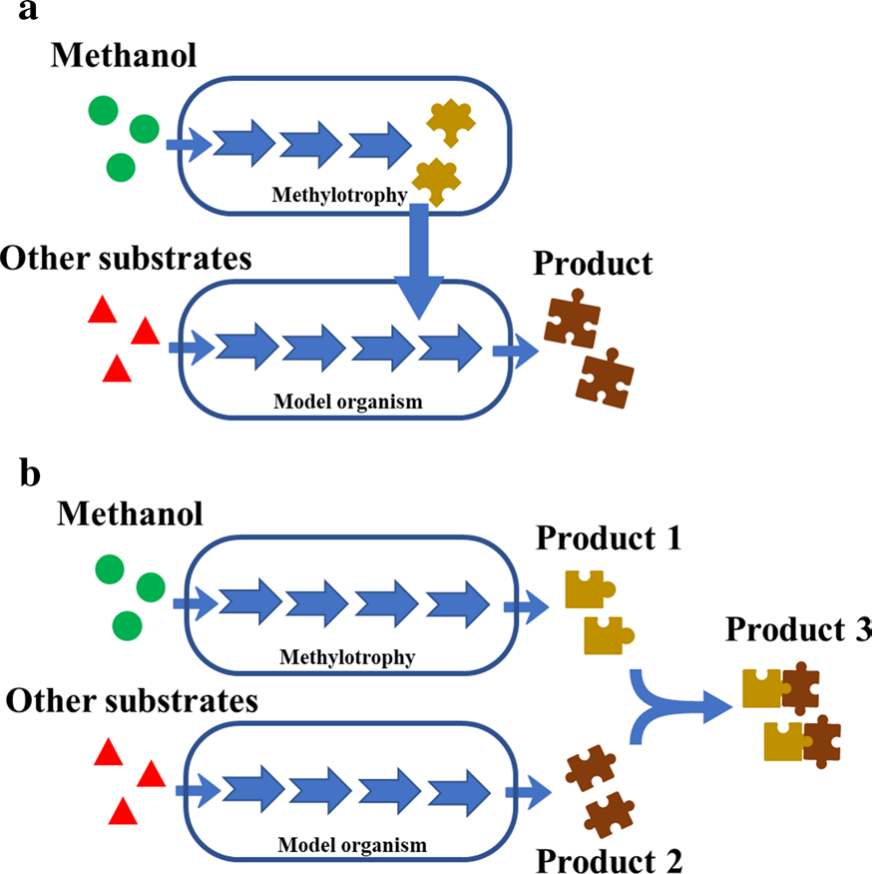
Schematics of the metabolic interaction of the methylotrophy and model organism consortium. **a** Commensalism in synthetic microbial consortia. **b** Cooperation in synthetic microbial consortia

Compared to genetically constructed methanol users, native methylotrophs show fast growth rates using methanol as sole carbon source, while only limited valuable metabolites can be produced due to the lack of efficient genetic tools for strain modification. Microbial consortia provide a new way to produce various chemicals from methanol. Two strategies were proposed for designing and constructing synthetic microbial consortia. One strategy is based on commensalism. In this case, elements and modules with different functions, such as protein scaffold elements or products synthesis modules, can be built in different strains, reducing the metabolic load on the single strain. The intermediate metabolites should be easily transported between the upstream and downstream strains under simultaneous co-culture condition (Fig. 3a). For example, methylotrophic bacteria were used to overproduce various amino acids from methanol, such as glutamate, lysine, threonine and serine, which can be further converted to other amino acids or derivatives, such as isoleucine, GABA and cadaverine, using engineered *E. coli* or other chassis microorganisms. The other strategy is based on cooperation. In this case, microbial consortia enable to rationally utilize different substrates and produce different products, in which one can consume methanol and the other can consume sugars under simultaneous co-culture condition (Fig. 3b). In addition, two kinds of products obtained from different microorganism can condense into a new product. For example, polyamide 56 (PA 56), a representative biopolymer, can be prepared by the polymerization of cadaverine with adipic acid [86]. Both monomers can be prepared using biological methods. As mentioned above, cadaverine can be produced from methanol by methylotrophic bacteria. Biosynthesis of adipic acid, an important platform chemical in industry, has also been realized in engineered *E. coli* [87]. Importantly, plenty of alkali was added to maintain the neutral conditions during microbial fermentation, increasing the osmotic pressure of the fermentation broth and inhibiting the performance of cell growth and metabolism [88]. Moreover, the cost of production and separation will increase. If a co-culture system is built, adipic acid could be condensed with cadaverine, which will change environment and improve cell growth and metabolism.

**Fig. 4:**
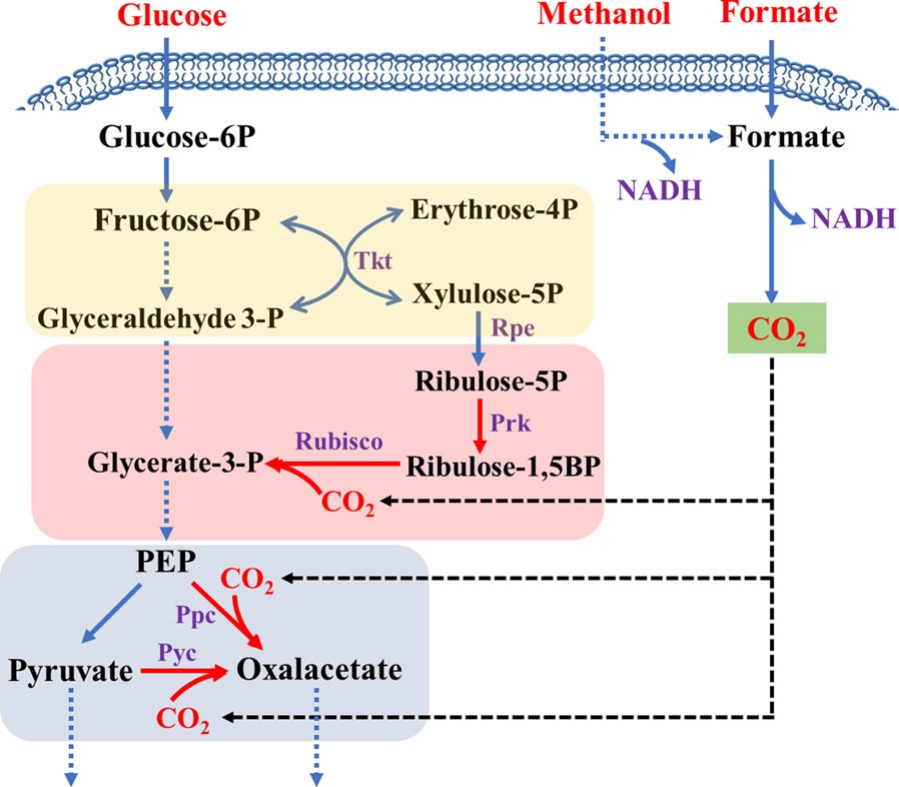
Decoupling glucose metabolism and carbon fixation in synthetic methylotrophy to achieve one carbon substrate effective utilization. Tkt, transketolase; Rpe, ribulose-5-phosphate 3-epimerase; Prk, phosphoribulokinase; Rubisco, ribulose bisphosphate carboxylase-oxygenase; Ppc, phosphoenolpyruvate carboxykinase; Pyc, pyruvate carboxylase

Compared to native methylotrophs, the key limitation of synthetic methylotrophs is the low efficiency of methanol utilization. On one hand, methanol dehydrogenases (MDHs), catalyzing methanol to formaldehyde, have poor thermodynamic properties. Screening new enzyme genes or modifying current enzymes can overcome this limitation. On the other hand, the replenishment of Ru5P/Xu5P is crucial to sustain the RuMP/XuMP pathway running. Overexpressing key genes of pentose phosphate pathway or finding appropriate promoters which are sensitive to the concentration of formaldehyde may be useful for overcoming this limitation. Actually, methanol assimilation was indeed improved by expressing of heterologous the non-oxidative pentose phosphate pathway from *B. methanolicus* [62]. Moreover, the strategy of synthetic protein scaffold or scaffoldless self-assembly system to cluster key enzymes of methanol assimilation can also improve methanol conversion [59]. Based on high degree of reduction in methanol and metabolic flux in methanol dissimilation pathway, a new way for efficiency of methanol metabolism was raised. In this case, methanol was firstly oxidized to formate and subsequently to CO_2_, generating a large number of reducing equivalents, which will benefit the production of reducing fuels and chemicals [89]. Moreover, if CO_2_ fixation pathways are introduced into synthetic methylotrophs, such as reductive TCA cycle or Calvin-Benson-Bassham pathway, the carbon loss problem will be solved (Fig. 4). However, we need also consider about energy conservation, redox balance and modules compatibility. Importantly, the other attractive one carbon compound, formate could also be used as co-carbon source.

## Conclusions

Methanol is increasingly becoming an attractive substrate for the production of various chemicals owning to its low-cost and renewability. A methanol-based bioeconomy has been proposed, in which a variety of chemicals were produced from methanol via biological fermentation. As reviewed above, challenges still exist ahead of development of methanol-based bioeconomy. However, with the matureness of biological technologies, such as metabolic engineering, protein engineering and synthetic biology, methanol utilization and products yield will be improved. It might be seeing more commodity chemicals from methanol in the near future.
